# Flip Chart based on the Interactive Theory of Breastfeeding: development and validation of an educational technology

**DOI:** 10.1590/0034-7167-2025-0046

**Published:** 2026-07-10

**Authors:** Thamires Fontoura Cantalejo, Candida Caniçali Primo, Nayara Christina Barbosa Pereira, Camila dos Santos Cotta, Míriam Carmo Rodrigues Barbosa, Dulce Maria Pereira Galvão, Eliane de Fatima Almeida Lima

**Affiliations:** I Universidade Federal do Espírito Santo. Vitória, Espírito Santo, Brazil; II Escola Superior de Enfermagem de Coimbra. Coimbra, Portugal

**Keywords:** Educational Technology, Validation Study, Breast Feeding, Nursing Theory, Health Education., Tecnología Educacional, Estudio de Validación, Lactancia Materna, Teoría de Enfermería, Educación en Salud.

## Abstract

**Objectives:**

to develop and validate a breastfeeding flip chart based on the Interactive Theory of Breastfeeding.

**Methods:**

cross-sectional, observational, quantitative study conducted in three phases: 1) organization of the theoretical content; 2) flip chart development; 3) evaluation by experts and the target audience.

**Results:**

the breastfeeding flip chart was organized into 27 pages presented as double-sided sheets. Sixteen experts rated it positively, with agreement exceeding 95.5%. In face validation with 101 pregnant and postpartum women, the technology was deemed appropriate, yielding a total Face Validity Index of 0.94, considered excellent.

**Final Considerations:**

experts and the target audience evaluated and approved the breastfeeding flip chart. This dialogue-based educational tool utilizes simple, clear language and features attractive illustrations and layout. It can be used to support health education delivered by healthcare professionals who care for pregnant and postpartum women.

## RESUMEN

**Objetivos:**

elaborar y validar un álbum seriado sobre lactancia materna basado en la Teoría Interactiva de la Lactancia.

**Métodos:**

estudio transversal, observacional y cuantitativo en tres etapas: 1) organización del contenido teórico; 2) desarrollo del álbum seriado; 3) evaluación por especialistas y público objetivo.

**Resultados:**

el Álbum Seriado sobre Lactancia Materna se organizó en 27 páginas como láminas a doble cara. Dieciséis especialistas lo aprobaron con >95,5% de acuerdo. En la evaluación de apariencia con 101 gestantes y puérperas, la tecnología se consideró adecuada y obtuvo un Índice de Validez de Apariencia total (IVA-T) de 0,94, clasificado como excelente.

**Consideraciones Finales:**

el Álbum Seriado sobre Lactancia Materna fue evaluado y aprobado por especialistas y público objetivo; es una herramienta dialógica, de lenguaje simple y claro, con ilustraciones y diseño atractivos, utilizable en la educación para la salud por profesionales que atienden a gestantes y puérperas.

## INTRODUCTION

Breastfeeding is a complex phenomenon that entails dynamic interaction between the mother-infant dyad and the environment, shaped by biological, psychological, sociocultural, policy, and economic factors^(1)^. Multiple factors hinder breastfeeding initiation and continuation-including insufficient support, cultural beliefs, economic barriers, and suboptimal practices within healthcare services, which contribute to low exclusive breastfeeding (EBF) rates worldwide and early weaning^(2,3)^.

According to the World Health Organization (WHO), the global EBF rate among infants younger than 6 months is 40%, and only 23 countries have achieved at least 60% of infants exclusively breastfed. These low rates are most prevalent in the Americas and Europe: only 6% of countries in the Americas report an EBF rate above 60%, whereas 25% of European countries have reached that level^(4-6)^.

In Brazil, 45.7% of children younger than 6 months are exclusively breastfed, whereas the global target is to reach at least 70% by 2030(6). The WHO recommends a combination of evidence based interventions to improve breastfeeding practices, including health education on the benefits and management of breastfeeding during prenatal and postpartum visits for pregnant and postpartum women and their families^(5-7)^.

Breastfeeding education and support provided by qualified healthcare professionals positively influence women’s attitudes toward initiating breastfeeding^(7-9)^. Educational technologies can strengthen health education initiatives that foster dialogue between service users and healthcare professionals^(10,11)^.

Although diverse educational technologies-such as educational videos, booklets, cordel literature, and flip charts-are widely used to promote breastfeeding, gaps remain regarding their effectiveness and their fit to the needs of the target audience^(12-16)^. In particular, the flip chart stands out for its simple, clear language and easily understood illustrations, which facilitate learning and strengthen rapport between healthcare professionals and the target audience^(10,16)^.

During the development of educational technologies, it is important to assess face validity with the target audience. This assessment examines aspects such as color palette, shapes, and the number and size of illustrations. It also evaluates whether the illustrations are clear, enable the content to be readily understood by the target audience, and align with the text, thereby supporting comprehension of the topic addressed by the technology^(15-17)^.

In light of low EBF rates and the need for effective health education strategies, this study aimed to develop and validate an educational technology grounded in an internationally recognized breastfeeding theory^(1)^. The objective is to address the elements that influence breastfeeding and contribute to health education efforts, promoting better practices for initiation and maintenance.

## OBJECTIVES

To develop and validate a breastfeeding flip chart based on the Interactive Theory of Breastfeeding.

## METHODS

### Ethical aspects

The study followed national and international ethical guidelines and was approved by the Research Ethics Committee. The Informed Consent Form (ICF) was obtained from all participants and signed in duplicate (two printed copies).

### Study design, period, and setting

Cross-sectional, observational, quantitative study to assess the content and face validity of an educational technology. This research is part of a larger project to implement the Baby-Friendly Hospital Initiative (BFHI) at a large university hospital in the state of Espírito Santo, in Brazil’s Southeast Region. Reporting adhered to the Strengthening the Reporting of Observational Studies in Epidemiology (STROBE) statement.

The study was conducted from February to June 2023 and comprised three steps: 1) organization of the theoretical content; 2) development of the flip chart; and 3) evaluation by experts and the target audience.

The flip chart is an educational technology consisting of bound panels arranged in sequence, with illustrations and brief texts; healthcare professionals use it to guide and raise awareness among the target audience about specific topics^(10,16)^. In this study, the Breastfeeding Flip Chart is intended for nurses or other healthcare professionals who provide care to pregnant and postpartum women (target audience) in outpatient or inpatient settings.

### Population/sample; inclusion and exclusion criteria

To organize the theoretical content and conduct the expert review, we recruited a convenience sample of 16 experts in maternal and child health. Inclusion criteria were: clinical staff with experience in breastfeeding care who worked in the maternity unit, Human Milk Bank (HMB), prenatal clinic, or neonatal intensive care unit (NICU); and faculty in women’s, child, and adolescent health affiliated with the institution.

A contracted designer laid out the flip chart and developed the illustrations, using the CuidarTech^®^ registered character family.

Face validation of the flip chart was conducted with pregnant and postpartum women admitted to the hospital’s maternity unit. We included postpartum women with liveborn infants who were hospitalized in the rooming-in unit within 6-48 hours postpartum, and pregnant women admitted for clinical treatment. We excluded women with psychological and/or psychiatric diagnoses of postpartum depression, mood disorders, or neurologic diseases, as well as postpartum women with medical contraindications or restrictions to breastfeeding, as documented in the medical record.

For the face validation stage, the sample size was calculated from the maternity unit’s average monthly admissions, considering a 95% confidence interval (95% CI) and a 5% margin of error. The final sample comprised 101 participants. Participants were randomly selected through direct, individual approaches on the inpatient wards.

### Study protocol

To develop the flip chart’s theoretical content, we used publications from the Brazilian Ministry of Health and the WHO, as well as articles presenting best-practice recommendations for breastfeeding term, preterm, and low-birth-weight infants^(3-9,18-23)^.

The flip chart pages were organized according to the 11 concepts of the Interactive Theory of Breastfeeding: dynamic interaction between mother and infant; the woman’s biological conditions; the infant’s biological conditions; the woman’s perception of breastfeeding; the child’s perception of breastfeeding; the woman’s body image; space for breastfeeding; maternal role; organizational systems for breastfeeding protection, promotion, and support; family and social authority; and the woman’s decision-making^(1)^.

To organize the theoretical content, we held an in-person meeting with the expert team working in the HMB, maternity unit, NICU, and outpatient clinic. The professionals selected content from best-practice sources and mapped it to the 11 concepts of the Interactive Theory of Breastfeeding, based on the theory’s concept definitions and content relevance. We then determined the sequence of the flip chart pages^(1)^.

In the expert review stage, the flip chart (front cover, back cover, illustrations, and facilitator guide sheets) was assessed for the material’s objectives; organization (distribution of content across the theory’s concepts); language and writing style; and the appearance of the images. Each item offered two response options: 1) Agree and 2) Disagree. A space for suggestions was provided below each flip chart page. The flip chart and the assessment instrument were emailed to the 16 maternal and child health experts, who returned the materials with suggested adjustments. Text edits and content reorganization were implemented, and the designer refined the images and layout.

Next, the target audience (pregnant and postpartum women) assessed the face validity of the prototype using IVATES (Appearance Validation Instrument for Health Educational Technologies). This instrument is validated in Portuguese and is widely used to evaluate educational technologies regarding appearance, clarity, and illustration relevance, as well as whether illustrations aid content comprehension^(17)^.

IVATES contains 12 items covering the following aspects: objective, organization, writing style, appearance, and motivation. Each item was rated on a five-point Likert scale: 1) Strongly disagree, 2) Disagree, 3) Neither agree nor disagree, 4) Agree, and 5) Strongly agree^(17)^.

The instrument retained its original structure and assessment format, with minor wording adjustments to improve comprehension among pregnant and postpartum women. These changes enabled participants to interpret items about the breastfeeding flip chart clearly, without altering the instrument’s core evaluative criteria.

To characterize participants in this stage, we conducted interviews using a Google Forms^®^ questionnaire to collect socioeconomic data (age, marital status, educational attainment, profession/occupation, household income in minimum wages, number of household residents, and race/skin color), obstetric history (weeks of gestation or the infant’s gestational age and number of live births), and breastfeeding experience (whether they had breastfed or lived with someone close who had breastfed; how long they breastfed; whether they had previously received information on breastfeeding and through which sources; whether they planned to breastfeed and for how long).

At the end of the questionnaire, two open-ended questions invited suggestions or critiques regarding the flip chart: “What did you think of the flip chart, its illustrations, and the topics covered?” and “Would you change or add anything to the flip chart?”.

Before data collection, a pilot study was conducted to refine the instruments and study procedures. The pilot lasted one month, was carried out on consecutive weekdays (Monday through Friday), and included 30 volunteers. After this stage, the necessary adjustments were implemented, and the study proceeded.

Face validation with the target audience took place from May to June 2023 on consecutive weekdays. It was conducted by the researcher alongside a team of 12 volunteers (undergraduate nursing students) who received 8 hours of training in instrument administration.

Pregnant and postpartum women were approached individually, and the study objectives and procedures were explained. After reading the Informed Consent Form (ICF), they signed it. Each participant then received a copy of the flip chart that contained only the illustration pages intended for the target audience.

We provided this partial copy to ensure that participants focused exclusively on the pages intended for them, since the content pages were designed solely for the professionals who administer the flip chart.

The research team then left the ward, allowing 15-20 minutes for participants to review the flip chart and complete the instrument. This approach was intended to make participants more comfortable as they examined the material, discussed it with a support person if present, and completed the face-validation instrument.

After this interval, to characterize the sample’s sociodemographic profile, a researcher administered the previously described interview using Google Forms^®^ on a cell phone. Each interview lasted up to 10 minutes. The total data collection time per participant was 30-40 minutes.

### Data analysis and statistics

Data were double-entered in Microsoft Excel (version 365) and analyzed in IBM SPSS Statistics, version 20.0. We performed a descriptive analysis of the interview data, calculating measures of central tendency and frequency distributions as part of the statistical analysis.

For face validation, we used the Face Validity Index (FVI). The item-level FVI (FVI-I) was computed as the number of participants who selected 4 or 5 divided by the total number of participants. The total FVI (FVI-T) was calculated as the sum of item-level FVI values divided by the number of items. Items with FVI ≥ 0.78 and FVI-T ≥ 0.90 were considered acceptable and required no revision^(17)^.

## RESULTS

**Figure 1:**
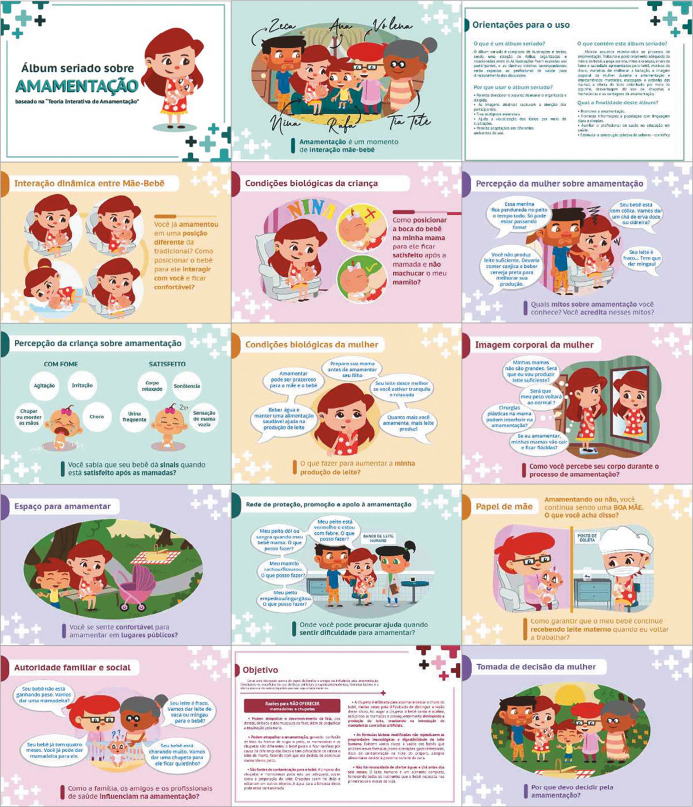
Panels of the Breastfeeding Flip Chart based on the Interactive Theory of Breastfeeding, Vitória, Espírito Santo, Brazil, 2023

In its final version, the Breastfeeding Flip Chart was produced on A3 paper, with all pages laminated and spiral-bound to facilitate handling by healthcare professionals. The material is available free of charge on the website: www.cuidartech.com.br. It was organized into 27 pages arranged as double-sided, full-color illustrated sheets, including a cover, an introductory page, 11 facilitator guide sheets containing guidance for professionals, and the corresponding 11 illustrations for the target audience. A technical sheet and a cataloging record were also included (Figure 1).

Each panel includes an illustrated page for the target audience that presents one concept from the theory^(1)^ and uses questions and illustrations to prompt discussion. On the reverse, intended for the professional who uses the flip chart, there is a guide outlining the content to be discussed with the audience. This guide specifies the main objective, key points, and evidence-based practices.

Panel 1 shows Ana in different breastfeeding positions: side-lying, cradle, koala (upright straddle), and football (clutch). It addresses the concept of interaction between mother and infant. The guide describes how to perform these positions and the key points for proper positioning of the mother and the infant. Panel 2 depicts Ana breastfeeding Nina, highlighting latch during feeding. It addresses the infant’s biological conditions. The guide outlines the key points for proper latch, signs of an ineffective latch, and positions that facilitate latch in preterm infants.

Panel 3 shows Ana holding Nina beside Zeca and explores common myths and beliefs about breastfeeding that shape women’s perceptions of this stage. Next, Panel 4 depicts Nina’s hunger and satiety cues and discusses the infant’s perception of breastfeeding and reasons for crying, so that women can distinguish these signals.

Panel 5 addresses the woman’s biological conditions, illustrated by Ana reflecting on questions about milk production. The text presents different factors that can improve lactation. Panel 6 covers the concept of the woman’s body image in relation to breastfeeding. The text discusses breast surgery, breast sagging/loss of firmness, and pre-pregnancy weight, and it shows Ana looking at herself in the mirror and her concerns about her body.

Panel 7 shows Ana breastfeeding in a public place (a park). It informs women of their right to breastfeed wherever they feel comfortable and safe. Panel 8 depicts Ana, accompanied by her husband Zeca, asking nurse Flora about possible breast complications. On the reverse, it presents key measures to relieve signs and symptoms of breast engorgement and cracked nipples, introduces the Human Milk Bank as part of the network for breastfeeding protection, promotion, and support, and provides step-by-step instructions for breast massage and hand expression.

Panel 9 shows Ana performing hand expression with Aunt Tetê beside her, while Lena uses a cup to offer Nina expressed breast milk. It addresses the challenge of balancing work and breastfeeding, explaining how to store expressed milk and how to offer it to the infant. It also discusses the maternal role, whether or not the woman breastfeeds.

The image in Panel 10 features Grandma Lena and Aunt Tetê discussing practices that negatively affect breastfeeding, such as the use of artificial nipples and bottles. The guide explains reasons for discouraging artificial nipples and the harms of introducing water, teas, and infant formula too early. This panel is intended to prompt discussion about family and social authority in breastfeeding.

Finally, the illustration in Panel 11 shows the whole family supporting Ana in breastfeeding. It addresses the benefits of breastfeeding for the infant, the woman, and society.

The expert evaluation was conducted by a multidisciplinary team of 14 nurses (5 PhD prepared faculty members, 6 nurses with a master’s degree in nursing, and 3 specialists), 1 nutritionist who was a master’s student, and 1 obstetrician with a PhD in public health. Participants were 24-58 years old; all were women; 93.75% had more than 10 years of clinical experience; and 87.5% self-identified as white.

All panels (cover, back cover, illustrations, and facilitator guide sheets) achieved more than 95.5% agreement across all evaluation items, with some suggestions for improvement.

In the face validation stage with the target audience, 101 pregnant and postpartum women participated. Of these, 84% were 20-39 years old, and 51% self-identified as brown (parda). In total, 86% reported having a partner (in a romantic relationship). Regarding education, 49% reported completing high school, and none reported being illiterate. With respect to employment, 52% reported paid work, 43% were unemployed or homemakers, and 5% were students. For household income, measured in minimum wages (set at BRL 1,300.00), 66% reported up to two minimum wages. In addition, 46% reported sharing their home with three to five residents, and 59.6% stated that their partner was the primary income earner.

Regarding obstetric history and breastfeeding experience, 38% of participants were in their first pregnancy, 28% were in their second, 19% reported having more than three children, and 15% reported having three living children. Among those who already had children, 88.4% had breastfed and 11.6% had not. Of those who breastfed, 32.3% breastfed for more than 12 months up to 24 months; 29.2% for more than 24 months; 18.5% for less than 6 months; 10.8% for 6-12 months; and 3.1% up to the child’s first 6 months of life.

When asked whether they had taken part during pregnancy in any course/lecture/discussion group on breastfeeding, 80.8% answered that they had not participated.

Regarding plans to breastfeed their children, 97% stated that they intend to breastfeed. Of these, 37.1% planned to breastfeed until natural weaning, 24.7% planned to breastfeed for more than 6 months up to 12 months, 13.4% for at least 6 months, 9.3% for more than 12 to 24 months, 9.3% for more than 24 months, 5.2% for less than 6 months, 1% until they return to work, and 3% reported that they did not wish to breastfeed.

Of the 101 participants, 72% were postpartum women; among these, 88.9% were hospitalized in the rooming-in unit with their infants, who were born at term (37-42 weeks). The remaining 28% were pregnant; 74.1% were in the third trimester (27-41 weeks).

In the face validation with pregnant and postpartum women, all 12 items were rated as adequate (Table 1), yielding a total Face Validity Index (FVI-T) of 0.94, considered excellent^(18)^.

**Table 1:** Face validation of the Breastfeeding Flip Chart based on the Interactive Theory of Breastfeeding, conducted with pregnant and postpartum women (target audience, N = 101), Vitória, Espírito Santo, Brazil, 2023

Items	1 Strongly disagree	2 Disagree	3 Neither agree nor disagree	4 Agree	5 Strongly agree	FVI
1) Are the illustrations appropriate for use with pregnant and breastfeeding women?	0	0	4	4	89	0.94
2) Are the illustrations clear and easy to understand?	1	0	0	3	89	0.96
3) Are the illustrations important for understanding the content presented?	2	1	0	3	89	0.95
4) Are the colors used in the illustrations appropriate for this type of material?	0	0	2	3	88	0.97
5) Are the shapes of the illustrations appropriate for this type of material?	0	0	1	1	88	0.96
6) Do the illustrations portray the everyday lives of breastfeeding women?	2	0	0	5	88	0.97
7) Is the layout of the illustrations on each page aligned with that page’s content?	1	1	2	5	88	0.93
8) Do the illustrations help make the flip chart content easier to understand?	0	0	3	1	88	0.95
9) Do the illustrations support the presentation of the topic and follow a logical sequence?	0	0	4	9	89	0.93
10) Is the number of illustrations in the flip chart appropriate?	0	0	6	8	89	0.92
11) Is the size of the illustrations appropriate?	0	0	4	3	89	0.95
12) Do the illustrations help change the behavior and attitudes of women who are breastfeeding or preparing to breastfeed?	0	0	7	8	89	0.91
TOTAL						0.94

In the qualitative assessment based on two open-ended interview questions, participants reported that the flip chart was engaging, highly illustrative, and suitable for individuals who cannot read. They noted that it addresses important content, and many said they were unfamiliar with some topics. Several participants emphasized the importance of making this technology available across all health services, including prenatal outpatient clinics, and said they would like to take the material home. Most stated that they would not change or add anything and that the flip chart was suitable for use.

On the other hand, some participants said they missed having more explanations and information in the illustrations. Accordingly, we reinforced that a flip chart is designed as a support technology for the professional, meaning that the content is presented and discussed by the professional responsible for dialogue and communication with the target audience.

## DISCUSSION

Educational technologies are strategic tools for health promotion because they strengthen health education by bringing professionals and patients together, facilitating knowledge exchange^(10,12,15)^. Educational interventions increase adherence, knowledge, positive attitudes, and maternal breastfeeding self-efficacy among women who participate in prenatal breastfeeding programs^(7-9,11,16)^.

The Breastfeeding Flip Chart, based on the Interactive Theory of Breastfeeding^(1)^, is an educational resource that supports and encourages this practice through dialogue and illustrations, with up-to-date, evidence-based content. It is innovative and distinct from other technologies because it is grounded in a middle-range theory that encompasses multiple factors influencing the breastfeeding process in diverse settings and is applicable across contexts^(10,16)^.

Breastfeeding success depends on multiple determinants, including a woman’s living conditions, return to work, social and cultural context, the presence or absence of a support network, and overcoming physical and emotional challenges^(2,3,22-24)^. Nevertheless, a societal narrative persists that places primary responsibility for breastfeeding and infant feeding on women, without acknowledging the complexity of this practice^(25)^.

This flip chart can help healthcare professionals address the range of factors that shape breastfeeding, including the woman’s body image while breastfeeding, the maternal role, the support network, family and social authority, and the woman’s decision-making^(1,26-28)^. These dimensions are seldom addressed, whereas most available educational materials focus on the biological conditions of the woman and the infant, clinical management, and myths and difficulties related to breastfeeding^(10-16,29)^.

Using the best available scientific evidence is essential when developing technologies. Doing so improves patient outcomes and the economic efficiency of health systems^(30)^.

Involving subject-matter experts in the development and evaluation of health educational technologies helps ensure that content adheres to ethical principles, avoids bias or approaches that could cause discrimination, and is tailored to the audience’s specific needs in their cultural context; experts also assess whether the content and purpose are appropriate^(13-15,31)^.

The target audience evaluated the flip chart positively: the illustrations were considered attractive, clear, and relevant to the topic, aiding comprehension. The assessment yielded an FVI-T of 0.94, considered excellent. This finding aligns with other studies in which target audiences assessed health educational technologies as objective, well-organized, understandable, engaging, and motivating^(31-34)^.

Illustrations that reflect the environments and situations experienced by the target audience capture attention^(31,32,34)^ and help visualize the ideas and themes addressed. Breastfeeding involves situations and myths transmitted across generations^(35)^. Therefore, enabling the audience to visualize these situations fosters a more attentive, critical perspective and supports the technology’s cultural and contextual fit.

The Breastfeeding Flip Chart, based on the Interactive Theory of Breastfeeding, can be used across care settings for pregnant and postpartum women to provide information in clear, simple language. In health education practice, it serves as a tool for professionals to co-construct knowledge and promote breastfeeding.

### Study limitations

This study has limitations. The technology was evaluated by healthcare professionals and the target audience from a single public institution, which limits the generalizability of the findings. Further clinical evaluation is needed to determine the effects of using the flip chart with the target audience.

### Contributions to nursing, health, or public policy

The flip chart supports nurses and other maternal and child health professionals in counseling pregnant and postpartum women about breastfeeding, using evidence-based content and official documents in an engaging, visually appealing format. It is a portable, easy-to-use, low-cost educational technology, making it an excellent option for adoption in public healthcare and teaching settings.

## CONCLUSIONS

The final version of the Breastfeeding Flip Chart, based on the Interactive Theory of Breastfeeding, contains 27 pages. It addresses topics related to the breastfeeding process, including: proper positioning of the mother and infant; proper latch; myths and beliefs about breastfeeding; signs of infant hunger and satiety; ways to improve lactation; the woman’s body image during and after breastfeeding; the maternal role; family and social authority in breastfeeding; breast complications; offering expressed breast milk; disadvantages of pacifier and bottle use; and the advantages of breastfeeding.

Sixteen experts evaluated the flip chart, achieving 95.5% agreement across all items related to the material’s objectives, organization (distribution of content across the theory’s concepts), writing style, and image appearance.

The material was also evaluated by pregnant and postpartum women, yielding a total Face Validity Index (FVI-T) of 0.94, which is considered excellent. All items achieved FVI ≥ 0.78, indicating the clarity and relevance of the content and illustrations.

We highlight the contributions of the multidisciplinary team, which helped select the content and develop the technology, adopting accessible language and practical guidance aligned with the best scientific evidence and official guidelines. We also underscore the work of the graphic designer, who created clean, approachable artwork with simple lines, soft colors, and characters representing different genders, ages, and ethnicities, enhancing representation.

The flip chart provides nurses and other healthcare professionals with an educational technology that fosters dialogue with pregnant and postpartum women, enhancing discussion and knowledge exchange about breastfeeding. It is feasible for practical use by healthcare professionals who care for pregnant and postpartum women, as well as across other levels of care and in teaching institutions. Future studies should assess the effectiveness of its use with the target audience.

## Data Availability

The research data are available within the article.
