# Supplementary material for: Flip Chart based on the Interactive Theory of Breastfeeding: development and validation of an educational technology
Source: Rev Bras Enferm. 2026 Jul 10;79:e20250046. doi: 10.1590/0034-7167-2025-0046 (PMC13363300; doi:10.1590/0034-7167-2025-0046)
Supplement: Translation

# Translation: Álbum Seriado baseado na Teoria Interativa de Amamentação: elaboração e validação de tecnologia educacional

**DOI:** 10.1590/0034-7167-2025-0046pt

**Authors:** Thamires Fontoura Cantalejo, Candida Caniçali Primo, Nayara Christina Barbosa Pereira, Camila dos Santos Cotta, Míriam Carmo Rodrigues Barbosa, Dulce Maria Pereira Galvão, Eliane de Fatima Almeida Lima

**Affiliations:** I Universidade Federal do Espírito Santo. Vitória, Espírito Santo, Brasil; II Escola Superior de Enfermagem de Coimbra. Coimbra, Portugal

**Keywords:** Tecnologia Educacional, Estudo de Validação, Amamentação, Teoria em Enfermagem, Educação em Saúde.

## Abstract

**Objetivos:**

elaborar e validar um álbum seriado sobre amamentação baseado na Teoria Interativa de Amamentação.

**Métodos:**

estudo transversal, observacional, de abordagem quantitativa, que seguiu as etapas: 1) Organização do conteúdo teórico; 2) Desenvolvimento do álbum seriado; 3) Avaliação por especialistas e público-alvo.

**Resultados:**

o Álbum Seriado sobre Amamentação foi organizado em 27 páginas, dispostas em lâminas frente e verso. Os 16 especialistas aprovaram o álbum com mais de 95,5% de concordância. Na avaliação da aparência com as 101 gestantes e puérperas, a tecnologia foi considerada adequada, obtendo um Índice de Validade de Aparência total de 0,94, considerado excelente.

**Considerações Finais:**

o Álbum Seriado sobre Amamentação foi avaliado e aprovado pelos especialistas e pelo público-alvo. É uma ferramenta dialógica, com linguagem simples e clara; desenhos e layout atrativos. Pode ser utilizado na promoção da educação em saúde por profissionais de saúde envolvidos na assistência a gestantes e puérperas.

## INTRODUÇÃO

Amamentar é um fenômeno complexo, que envolve um processo de interação dinâmica entre o binômio mãe-filho e o ambiente, com influência de questões biológicas, psicológicas, socioculturais, políticas e econômicas^(1)^. Diversos fatores dificultam o início e a continuidade da amamentação, como a falta de suporte adequado, crenças culturais, barreiras econômicas e práticas inadequadas nos serviços de saúde, o que contribui para as baixas taxas de amamentação exclusiva no mundo e desmame precoce^(2,3).^

Dados da Organização Mundial da Saúde (OMS) mostram que as taxas de amamentação exclusiva em bebês menores de 6 meses de idade são atualmente de 40%, e apenas 23 países alcançaram pelo menos 60% de recém-nascidos amamentados exclusivamente. Essas baixas taxas são predominantes nas Américas e na Europa: apenas 6% dos países das Américas registraram uma taxa de amamentação exclusiva superior a 60%, ao passo que 25% dos países europeus alcançaram essa cifra^(4-6)^.

No Brasil, 45,7% das crianças com menos de 6 meses são amamentadas de forma exclusiva, enquanto a meta global é alcançar pelo menos 70% até 2030^(6)^. A OMS recomenda uma combinação de intervenções baseadas em evidências destinadas a melhorar as práticas de amamentação, que inclui educação em saúde sobre os benefícios e manejo da amamentação nas consultas de pré-natal e no pós-parto para gestantes, puérperas e suas famílias^(5-7)^.

A educação e o apoio sobre a amamentação prestados por profissionais de saúde qualificados influenciam positivamente as atitudes das mulheres em relação ao início desse processo^(7-9)^. As tecnologias educacionais podem contribuir com as ações de educação em saúde que efetivam o diálogo entre usuários e profissionais de saúde^(10,11)^.

Embora diversos tipos de tecnologias educacionais - como vídeos educativos, cartilhas, literatura de cordel e álbuns seriados - sejam amplamente utilizados para promover a amamentação, ainda existem lacunas na avaliação de sua eficácia e adequação às necessidades do público-alvo^(12-16)^. Em particular, o álbum seriado destaca-se pela linguagem simples e clara e pelas ilustrações de fácil compreensão, que favorecem o aprendizado e fortalecem o vínculo entre os profissionais de saúde e o público-alvo^(10,16)^.

No processo de elaboração de tecnologias educacionais, é importante que sua aparência seja avaliada pelo público-alvo. Essa avaliação visa verificar aspectos relacionados às cores, formas, quantidade e tamanhos das ilustrações. Também avalia se as ilustrações são claras, se permitem que o conteúdo seja facilmente compreendido pelo público-alvo e se elas harmonizam-se com o texto, favorecendo a compreensão da temática abordada na tecnologia^(15-17)^.

Diante das baixas taxas de amamentação exclusiva e da necessidade de estratégias eficazes para educação em saúde, este estudo propôs-se a elaborar e validar uma tecnologia educacional fundamentada em uma teoria de amamentação reconhecida internacionalmente^(1)^. O objetivo é abordar os diversos elementos que influenciam o processo de amamentação e contribuir para as ações de educação em saúde, promovendo melhores práticas para a iniciação e manutenção desse processo.

## OBJETIVOS

Elaborar e validar um álbum seriado sobre amamentação baseado na Teoria Interativa de Amamentação.

## MÉTODOS

### Aspectos éticos

O estudo foi conduzido de acordo com as diretrizes éticas nacionais e internacionais e aprovado pelo Comitê de Ética em Pesquisa. O Termo de Consentimento Livre e Esclarecido foi obtido de todos os indivíduos envolvidos no estudo por meio de assinatura em duas vias impressas.

### Desenho do estudo, período e local do estudo

Estudo transversal, observacional, de abordagem quantitativa, para avaliação de conteúdo e aparência de uma tecnologia educacional. Esta pesquisa integra um macroprojeto de implementação da Iniciativa Hospital Amigo da Criança (IHAC) em um hospital universitário de grande porte no estado do Espírito Santo, Região Sudeste do Brasil. O relatório da pesquisa seguiu o *Strengthening the reporting of observational studies in epidemiology* (STROBE).

A pesquisa ocorreu de fevereiro a junho de 2023 e seguiu estas etapas: 1) Organização do conteúdo teórico; 2) Desenvolvimento do álbum seriado; 3) Avaliação por especialistas e pelo público-alvo.

O álbum seriado é uma tecnologia educacional composta por lâminas encadernadas ou em sequência, ilustradas e acompanhadas de textos curtos; é utilizada pelo profissional de saúde para orientar e sensibilizar o público-alvo sobre temas específicos^(10,16)^. Neste estudo, o Álbum Seriado sobre Amamentação é para ser utilizado pelo enfermeiro ou profissional de saúde que atua na assistência às gestantes ou puérperas (público-alvo) em cenário ambulatorial ou hospitalar.

### População ou amostra; critérios de inclusão e exclusão

Para a organização do conteúdo teórico e avaliação por especialistas, foram selecionados por conveniência 16 especialistas da área materno-infantil. Estes foram os critérios de inclusão: membros do corpo clínico com experiência em cuidado em amamentação e que atuassem na maternidade, Banco de Leite Humano (BLH), ambulatório de pré-natal ou Unidade de Terapia Intensiva Neonatal (UTIN); e docentes da área da saúde da mulher, criança e adolescente que atuassem na instituição.

Uma designer contratada diagramou e desenvolveu as ilustrações do álbum, utilizando a família de personagens registrada pelo CuidarTech^®^.

A avaliação da aparência do álbum seriado foi realizada com gestantes e puérperas internadas na maternidade do hospital. Foram incluídas puérperas com recém-nascidos vivos, internadas no alojamento conjunto, estando dentro do período de 6 a 48 horas pós-parto; e gestantes internadas em tratamento clínico. Foram excluídas da pesquisa as pacientes que apresentassem os diagnósticos psicológicos e/ou psiquiátricos de depressão pós-parto, transtornos de humor e doenças neurológicas, bem como puérperas com contraindicação e/ou restrição médica para amamentar, conforme registro em prontuário.

Para delimitação da amostra na etapa de avaliação da aparência, foi feito um cálculo com base no número de internações mensais na maternidade do hospital em estudo, considerando um intervalo de confiança de 95% e margem de erro de 5%. A amostra obtida foi de 101 participantes. As participantes foram escolhidas aleatoriamente por meio de abordagem direta e individual nas enfermarias.

### Protocolo do estudo

Para elaboração do conteúdo teórico do álbum seriado, foram utilizadas publicações do Ministério da Saúde e da OMS bem como artigos que traziam as recomendações das melhores práticas relacionadas à amamentação de recém-nascidos a termo, prematuros e de baixo peso^(3-9,18-23)^.

O conteúdo das lâminas do álbum foi organizado com base nos 11 conceitos da Teoria Interativa de Amamentação: interação dinâmica entre mãe e bebê; condições biológicas da mulher; condições biológicas do bebê; percepção da mulher sobre amamentação; percepção da criança sobre amamentação; imagem corporal da mulher; espaço para amamentar; papel de mãe; sistema organizacionais de proteção, promoção e apoio à amamentação; autoridade familiar e social; e tomada de decisão da mulher^(1)^.

Para organizar o conteúdo teórico, foi realizada uma reunião presencial com a equipe de especialistas que atuam no BLH, maternidade, UTIN e ambulatório. Os profissionais selecionaram o conteúdo dos materiais com as melhores práticas e distribuíram-no entre os 11 conceitos da Teoria Interativa de Amamentação, conforme as definições dos conceitos da teoria e a relevância dos conteúdos. Depois, foi feita a organização da sequência do álbum seriado^(1)^.

Na etapa de avaliação por especialistas, o álbum seriado (capa, contracapa, ilustrações e fichas-roteiro) foi analisado em relação aos objetivos do material, organização (distribuição dos conteúdos entre os conceitos da teoria), linguagem e estilo de escrita, bem como aparência das imagens. Cada item disponibilizava duas opções de avaliação: 1) Concordo e 2) Discordo. Abaixo de cada lâmina do álbum seriado, havia um espaço para sugestões. O álbum seriado e o instrumento foram enviados via e-mail para os 16 especialistas na área materno-infantil. O material foi devolvido via e-mail com as sugestões de adequações. As correções na escrita e reorganização dos conteúdos foram feitas, e a designer corrigiu as imagens e a diagramação.

Na sequência, o público-alvo (gestantes e puérperas) avaliou a aparência do protótipo por meio do instrumento IVATES (Instrumento de Validação de Aparência de Tecnologia Educacional em Saúde). Ele é validado na língua portuguesa e amplamente utilizado para avaliar tecnologias educacionais quanto à aparência, clareza e relevância das ilustrações, além de verificar se elas auxiliam na compreensão do conteúdo^(17)^.

O IVATES tem 12 itens, que abrangem os seguintes aspectos: objetivo, organização, estilo da escrita, aparência e motivação. Cada item foi avaliado mediante uma escala do tipo Likert com cinco opções de resposta: 1) Discordo totalmente, 2) Discordo, 3) Não concordo nem discordo, 4) Concordo e 5) Concordo totalmente^(17)^.

Esse instrumento manteve a estrutura e a forma avaliativa originais, com pequenas modificações na redação dos itens, visando facilitar a compreensão pelas gestantes e puérperas. Tais alterações garantiram que os participantes pudessem interpretar claramente as questões relacionadas a um álbum seriado sobre amamentação, sem comprometer a essência dos critérios avaliativos do instrumento original.

Para a caracterização das participantes dessa etapa, foram realizadas entrevistas utilizando um formulário (*Google Forms* ^®^) para coletar dados do perfil socioeconômico (idade, situação conjugal, nível de escolaridade, profissão/ocupação, renda familiar em salários mínimos, número de moradores no domicílio e raça/cor), história obstétrica (semanas de gestação ou idade gestacional do bebê e número de nascidos vivos) e experiência com amamentação (se já amamentou ou conviveu com alguém próximo que amamentou, por quanto tempo amamentou, se já recebeu informações sobre amamentação e por quais meios, se planeja amamentar e até quando).

Ao final do formulário, foram incluídas duas perguntas abertas para sugestões ou críticas das participantes quanto ao álbum: “O que você achou do álbum, das ilustrações e temas abordados?” e “Você mudaria ou acrescentaria algo no álbum seriado?”.

Antes da coleta de dados, foi conduzido um estudo-piloto para ajustar os instrumentos e a metodologia da pesquisa. O piloto teve duração de um mês, sendo realizado em dias consecutivos (de segunda a sexta-feira), e contou com a participação de 30 voluntários. Após essa etapa, as correções necessárias foram implementadas, permitindo o prosseguimento do estudo.

A avaliação da aparência do álbum seriado com o público-alvo transcorreu de maio a junho de 2023, em dias semanais consecutivos. Foi conduzida pela pesquisadora junto com uma equipe de 12 voluntárias, que eram graduandas de Enfermagem e haviam recebido treinamento de oito horas relacionado à aplicação dos instrumentos.

As gestantes e/ou puérperas eram abordadas individualmente, sendo-lhes explicados o objetivo e as etapas da pesquisa. Após a leitura do Termo de Consentimento Livre e Esclarecido, elas o assinavam. Em seguida, cada participante recebia um exemplar do álbum seriado que continha apenas as páginas ilustrativas direcionadas ao público-alvo.

A entrega desse exemplar incompleto objetivou garantir que o público-alvo focasse exclusivamente na avaliação das lâminas destinadas a ele. Isso se justifica pelo fato de as lâminas de conteúdo serem voltadas exclusivamente aos profissionais responsáveis pela aplicação do álbum.

As pesquisadoras se retiravam da enfermaria, dando um intervalo de 15 a 20 minutos para a participante manusear o álbum e responder ao instrumento. Essa ação visava deixar a participante mais confortável e à vontade para manusear o álbum, comentar com seu(sua) acompanhante sobre o material e responder ao instrumento de avaliação de aparência.

Após esse período, a fim de caracterizar o perfil sociodemográfico da amostra, a pesquisadora realizou a entrevista utilizando o *Google Forms* ^®^, em aparelho celular. Cada entrevista tinha duração de, no máximo, dez minutos. Todo o processo de coleta de dados durava entre 30 e 40 minutos com cada participante.

### Análise dos resultados e estatística

Os dados foram tabulados no programa Microsoft Excel (versão 365) em duas entradas e analisados no *software Statistical Package for the Social Sciences* (SPSS) 20.0. Foi realizada a análise descritiva dos dados coletados nas entrevistas, com cálculo de medidas de tendência central e distribuição de frequências.

Na avaliação da aparência, utilizou-se o Índice de Validade de Aparência (IVA). O IVA para cada item (IVA-I) foi computado pelo número de participantes que responderam 4 ou 5, dividido pelo total de participantes. Para o IVA total (IVA-T), foi realizada a soma dos IVA-I, dividida pelo total de itens. Consideraram-se aceitáveis, sem necessidade de correção, os itens com IVA igual ou superior a 78% (IVA ≥ 0,78) e IVA-T ≥ 0,90^(17)^.

## RESULTADOS

Em sua versão final, o Álbum Seriado sobre Amamentação foi formatado em papel sulfite, tamanho A3, com todas as páginas plastificadas e encadernadas em espiral para facilitar o manuseio pelos profissionais de saúde. O material está disponível gratuitamente neste website: www.cuidartech.com.br. Foi organizado em 27 páginas, dispostas em lâminas (páginas) frente e verso, coloridas e ilustrativas, incluindo capa, apresentação, 11 fichas-roteiro, com conteúdo de orientação ao profissional, bem como as respectivas 11 ilustrações, direcionadas ao público-alvo. Além disso, constavam a ficha técnica e a ficha catalográfica (Figura 1).

**Figura 1:** Lâminas do Álbum Seriado sobre Amamentação baseado na Teoria Interativa de Amamentação, Vitória, Espírito Santo, Brasil, 2023

Em cada lâmina, há uma página ilustrativa orientada ao público-alvo, que aborda e explica um dos conceitos da Teoria^(1)^, expondo perguntas e ilustrações que aproximam o público ao conteúdo proposto para discussão. No verso de cada página, voltado ao profissional que manuseia o álbum, há um roteiro do conteúdo a ser discutido com o público-alvo. Esse roteiro descreve os conteúdos a serem abordados: o objetivo principal, os pontos-chave e as práticas baseadas nas melhores evidências.

Na Lâmina 1, observa-se Ana realizando diferentes posições para amamentar: deitada, tradicional, cavalinho e invertida. É trabalhado o conceito de interação entre mãe e filho. Na ficha-roteiro, são descritos o modo de realização dessas posições e os pontos-chave para o posicionamento adequado da mãe e da criança. A Lâmina 2 apresenta Ana amamentando Nina, evidenciando a pega durante a amamentação. Aborda as condições biológicas da criança. O roteiro descreve os pontos-chave da pega adequada, os sinais indicativos de pega inadequada e as posições que favorecem a pega do bebê prematuro.

A Lâmina 3 exibe a ilustração de Ana segurando Nina ao lado de Zeca, com dúvidas sobre os mitos e crenças comuns durante a amamentação, que influenciam a percepção da mulher sobre essa fase. Em seguida, a Lâmina-roteiro 4 ilustra a Nina com sinais de saciedade e de fome, discutindo a percepção da criança sobre a amamentação, os sinais de fome, saciedade e os motivos do choro, para que a mulher aprenda a distingui-los.

A Lâmina 5 refere-se às condições biológicas da mulher, ilustrada por Ana pensando nos questionamentos sobre a produção de leite. O texto apresenta diferentes fatores para melhorar a lactação. A Lâmina 6 aborda o conceito de imagem corporal da mulher em relação ao processo de amamentar. O texto versa sobre as mamoplastias, flacidez das mamas e peso pré-gestacional. Ilustra a Ana observando-se em frente ao espelho e suas inseguranças sobre o corpo.

A Lâmina 7 apresenta Ana amamentando em um lugar público (parque). Visa orientar a mulher sobre o seu direito de amamentar em qualquer lugar em que ela se sinta confortável e protegida. A Lâmina 8 exibe Ana, acompanhada por seu marido Zeca, questionando a enfermeira Flora a respeito das possíveis intercorrências mamárias. No verso, aborda os principais cuidados para o alívio dos sinais e sintomas do ingurgitamento mamário e da fissura mamilar, bem como apresenta o banco de leite como um dos locais da rede de proteção, promoção e apoio à amamentação. Também descreve o passo a passo da massagem e ordenha das mamas.

A Lâmina 9 é composta pela imagem de Ana realizando a ordenha manual acompanhada da tia Tetê, enquanto Lena usa um copinho para ofertar a Nina o leite retirado. Apresenta o desafio de conciliar trabalho e amamentação, abordando como armazenar o leite ordenhado e a forma de ofertá-lo à criança. Também discute a questão do papel de mãe quando da realização ou não da amamentação.

A imagem da Lâmina 10 traz a avó Lena e a tia Tetê abordando práticas que interferem negativamente na amamentação, como o uso de bicos artificiais e mamadeiras. O roteiro expõe os motivos para o desencorajamento do uso de bicos artificiais e os malefícios da introdução precoce de água, chás e fórmulas lácteas. Essa lâmina tem a finalidade de gerar uma discussão acerca da autoridade familiar e social em relação à amamentação.

Por fim, a ilustração da Lâmina 11 apresenta toda a família junta apoiando Ana na amamentação. Trata dos benefícios da amamentação para a criança, para a mulher e para a sociedade.

A avaliação pelos especialistas foi realizada por uma equipe multiprofissional de 14 enfermeiras (5 doutoras e docentes, 6 mestres em enfermagem e 3 especialistas); 1 nutricionista mestranda; e 1 médica obstetra, doutora em saúde coletiva. Quanto à caracterização dos profissionais, a idade era de 24 a 58 anos, 100% eram do sexo feminino, 93,75% tinham mais de dez anos de experiência clínica, e 87,5% autodeclararam-se de cor branca.

Todas as lâminas (capa, contracapa, ilustrações e fichas-roteiro) do álbum seriado tiveram mais de 95,5% de concordância em todos os itens de avaliação, havendo algumas sugestões.

Na etapa de avaliação da aparência com o público-alvo, participaram 101 gestantes e puérperas, das quais 84% tinham entre 20 e 39 anos de idade, e 51% autodeclararam-se de cor parda. Do total, 86% referiram ter alguma parceria/relacionamento amoroso. Quanto à escolaridade, 49% disseram ter ensino médio completo, e nenhuma participante relatou ser analfabeta. No tocante à atividade remunerada, 52% afirmaram exercer alguma atividade, 43% estavam desempregadas ou eram do lar e 5% eram estudantes. Quanto à renda familiar, em salários mínimos (considerando o valor de R$ 1.300,00), 66% das participantes recebiam até dois salários mínimos. Ainda, 46% das participantes referiram dividir a casa com três a cinco moradores, e 59,6% declararam que a maior renda vinha do parceiro.

No que concerne à história obstétrica e experiência com amamentação, 38% das participantes estavam na primeira gestação, 28% estavam na segunda gestação, 19% declararam ter mais de três filhos e 15% disseram ter três filhos vivos. Das participantes que já tinham filhos, 88,4% afirmaram ter amamentado, e 11,6% não amamentaram. Dentre as participantes que amamentaram, 32,3% amamentaram por mais de 12 meses até 24 meses; 29,2% amamentaram por mais de 24 meses; 18,5%, por menos de 6 meses; 10,8%, entre 6 e 12 meses; e 3,1% amamentaram até 6 meses de vida da criança.

Quando questionadas se elas já haviam participado, ao longo da gestação, de algum curso/palestra/roda de conversa cujo tema fosse amamentação, 80,8% responderam não ter participado.

Em relação ao planejamento para amamentar seus filhos(as), 97% afirmaram que desejam amamentar. Destas, 37,1% relataram que planejam amamentar até o desmame natural, 24,7% planejam amamentar mais de 6 meses até 12 meses, 13,4% até pelo menos 6 meses, 9,3% mais de 12 a 24 meses, 9,3% mais de 24 meses, 5,2% por menos de 6 meses, 1% respondeu que amamentará até quando voltar a trabalhar, e 3% das participantes disseram que não desejavam amamentar seus filhos(as).

Das 101 participantes, 72% eram puérperas, com 88,9% dessas mulheres internadas no alojamento junto com seus filhos, que nasceram a termo (entre 37 e 42 semanas). Das participantes, 28% eram gestantes, das quais 74,1% estavam no 3° trimestre de gestação (entre 27 e 41 semanas).

Na avaliação da aparência com as gestantes e puérperas, todos os 12 itens foram considerados adequados (Tabela 1), obtendo-se um IVA-T de 0,94, que é considerado excelente^(18)^.

**Tabela 1:** Avaliação da aparência do Álbum Seriado sobre Amamentação baseado na Teoria Interativa de Amamentação, realizada pelas gestantes e puérperas (público-alvo, N = 101), Vitória, Espírito Santo, Brasil, 2023

Itens	1 Discordo totalmente	2 Discordo	3 Discordo parcialmente	4 Concordo	5 Concordo totalmente	IVA
1) As ilustrações estão adequadas para serem usadas com gestantes e mulheres que amamentam?	0	0	4	4	89	0,94
2) As ilustrações são claras e fáceis de compreender?	1	0	0	3	89	0,96
3) As ilustrações são importantes para que o conteúdo abordado seja entendido?	2	1	0	3	89	0,95
4) As cores utilizadas nas ilustrações estão adequadas para o tipo de material?	0	0	2	3	88	0,97
5) As formas das ilustrações estão adequadas para o tipo de material?	0	0	1	1	88	0,96
6) As ilustrações retratam o cotidiano das mulheres que amamentam?	2	0	0	5	88	0,97
7) A distribuição das figuras, em cada página, está em harmonia com o conteúdo a ser transmitido naquela página?	1	1	2	5	88	0,93
8) As figuras utilizadas tornam mais fácil de entender o conteúdo do álbum seriado?	0	0	3	1	88	0,95
9) As ilustrações auxiliam na exposição do tema e estão em uma sequência lógica (que faz sentido)?	0	0	4	9	89	0,93
10) As ilustrações estão em quantidade adequada no álbum seriado?	0	0	6	8	89	0,92
11) O tamanho das ilustrações está adequado?	0	0	4	3	89	0,95
12) As ilustrações ajudam na mudança de comportamento e atitude das mulheres que já amamentam ou se preparam para amamentar?	0	0	7	8	89	0,91
TOTAL						0,94

Na avaliação qualitativa por meio das duas perguntas abertas na entrevista, as participantes relataram que o álbum seriado é interessante e bem ilustrativo, sendo uma ferramenta adequada, também, para pessoas que não sabem ler. Elas apontaram que o álbum aborda conteúdos importantes, e muitas referiram não conhecer alguns assuntos. Além disso, várias participantes expressaram a importância de disponibilizar essa tecnologia em todos os serviços de saúde, incluindo os ambulatórios de pré-natal. Também disseram que gostariam de levar o material para casa. A maioria afirmou que não mudaria nem acrescentaria nada e que o álbum estava adequado para ser utilizado.

Por outro lado, algumas participantes relataram que sentiram falta de mais explicações e informações nas ilustrações. Nesse sentido, foi reforçado que um álbum seriado tem como objetivo ser uma tecnologia de apoio para o profissional, de modo que o conteúdo é apresentado e discutido pelo profissional encarregado do diálogo e comunicação com o público-alvo.

## DISCUSSÃO

Tecnologias educacionais constituem ferramentas estratégicas na promoção da saúde, pois potencializam o processo de educação em saúde ao integrar profissional e paciente, facilitando a intermediação do conhecimento^(10,12,15)^. O uso de intervenções educativas aumenta a adesão, o conhecimento, a atitude positiva e a autoeficácia materna em relação à amamentação entre mães que participam de programas educacionais sobre esse tema no pré-natal^(7-9,11,16)^.

O Álbum Seriado sobre Amamentação baseado na Teoria Interativa de Amamentação^(1)^ constitui um recurso didático que auxilia e incentiva essa prática de forma dialogada e ilustrativa, com conteúdo atualizado e fundamentado nas melhores evidências científicas. O álbum é inovador e diferenciado de outras tecnologias, pois fundamenta-se em uma teoria de médio alcance que engloba vários fatores capazes de influenciar o processo de amamentação em distintos cenários e que é aplicável a diferentes contextos^(10,16)^.

Compreende-se que o sucesso da amamentação está atrelado a múltiplos condicionantes: padrão de vida da mulher, retorno ao trabalho, contexto social e cultural em que está inserida, presença ou não de uma rede de apoio e superação das dificuldades físicas e emocionais^(2,3,22-24)^. No entanto, prevalece na sociedade um discurso de responsabilização da mulher sobre a amamentação e alimentação de seu filho, sem considerar toda essa complexidade da prática^(25)^.

Esse álbum seriado poderá auxiliar os profissionais de saúde a traduzirem a diversidade de fatores que influenciam o processo da amamentação, englobando questões relacionadas à imagem corporal da mulher que amamenta, ao papel de mãe, à rede de apoio, à autoridade familiar e social e à tomada de decisão da mulher^(1,26-28)^. Tais aspectos são pouco discutidos, enquanto a maioria dos materiais educativos disponíveis traz questões ligadas às condições biológicas da mulher e do bebê; ao manejo clínico da amamentação; e aos mitos e dificuldades para amamentar^(10-16,29)^.

O uso das melhores evidências científicas é primordial nos estudos de desenvolvimento de tecnologias. Essa utilização melhora os resultados dos pacientes e a eficiência econômica dos sistemas de saúde^(30)^.

A participação de profissionais especialistas na elaboração e avaliação de tecnologias educacionais na área da saúde possibilita que o conteúdo respeite princípios éticos, evitando vieses ou abordagens que possam causar discriminação; além disso, os profissionais avaliam se o material está adaptado às necessidades específicas do público em função do contexto cultural e se o conteúdo e o objetivo são adequados^(13-15,31)^.

O público-alvo avaliou de forma positiva o álbum: as ilustrações foram consideradas atrativas, claras e pertinentes ao tema, auxiliando na compreensão do conteúdo - o IVA-T foi de 0,94, que é classificado como excelente. Tal resultado corrobora aqueles encontrados em outros estudos de desenvolvimento e avaliação de tecnologias educacionais em saúde realizada pelo público-alvo, em que o material foi considerado objetivo, organizado, compreensivo, lúdico e motivador^(31-34)^.

Ilustrações que representam os ambientes e situações vivenciadas pelo público-alvo atraem a atenção^(31,32,34)^ e auxiliam na visualização das ideias e temas abordados. A prática da amamentação envolve situações e mitos transmitidos de geração em geração^(35)^. Portanto, possibilitar que o público-alvo visualize tais situações favorece um olhar mais atento e crítico, refletindo a adequação cultural e de contexto da tecnologia construída.

O Álbum Seriado sobre Amamentação baseado na Teoria Interativa de Amamentação poderá ser utilizado em diferentes contextos de assistência à gestante e puérpera, para fornecer informações com linguagem clara e simples. No processo de educação em saúde, será uma ferramenta para o profissional na construção de saberes e promoção da amamentação.

### Limitações do estudo

O estudo apresenta limitações: a avaliação da tecnologia foi conduzida com profissionais de saúde e público-alvo de uma única instituição pública, limitando a extrapolação dos resultados. Também é preciso realizar uma avaliação clínica que comprove os efeitos do uso do álbum seriado no público-alvo.

### Contribuições para área da Enfermagem, Saúde ou Política Pública

O álbum seriado apoia enfermeiros e demais profissionais de saúde da área materno-infantil no aconselhamento de gestantes e puérperas sobre a amamentação, com base em evidências científicas e documentos oficiais, em formato lúdico e atrativo. Além do mais, trata-se de uma tecnologia educativa portátil, de fácil manuseio e de baixo custo, sendo uma excelente ferramenta para incorporação nos ambientes públicos de saúde e ensino.

## CONCLUSÕES

A versão final do Álbum Seriado sobre Amamentação baseado na Teoria Interativa de Amamentação contém 27 páginas. Aborda assuntos relacionados ao processo de amamentação, tais como: posicionamento adequado da mãe e do bebê, pega correta, mitos e crenças relacionados à amamentação, sinais de fome e saciedade apresentados pelo bebê, maneiras de melhorar a lactação, imagem corporal da mulher durante e após a fase de amamentação, o papel de mãe, autoridade familiar e profissional na amamentação, intercorrências mamárias, oferta do leite ordenhado, desvantagem do uso de chupetas e mamadeiras e as vantagens da amamentação.

Os 16 especialistas avaliaram o álbum seriado com 95,5% de concordância em todos os itens relacionados aos objetivos do material, organização (distribuição dos conteúdos entre os conceitos da teoria), estilo de escrita e aparência das imagens.

O material foi avaliado também pelas gestantes e puérperas, obtendo Índice de Validade de Aparência total (IVA-T) de 0,94, considerado excelente. Todos os itens avaliados obtiveram IVA maior que 0,78, o que demonstra a clareza e relevância do conteúdo e ilustrações presentes na tecnologia.

Ressalta-se a contribuição da equipe multidisciplinar, que colaborou na seleção do conteúdo e construção da tecnologia, adotando uma linguagem acessível bem como orientações práticas alinhadas às melhores evidências científicas e em conformidade com diretrizes oficiais. Além disso, destaca-se a atuação da designer gráfica, responsável por ilustrações atrativas em arte limpa e amigável, com traços simples, cores suaves e personagens de diferentes gêneros, idades e etnias, reforçando a representatividade.

O álbum seriado busca proporcionar ao enfermeiro e profissional de saúde uma tecnologia educacional que suscita o diálogo com gestantes e puérperas, qualificando a discussão e troca de conhecimentos sobre amamentação. Mostra-se viável para uso prático por profissionais de saúde envolvidos na assistência a gestantes e puérperas, bem como em outros níveis de atenção à saúde e em instituições que promovam o ensino. Sugere-se que outros estudos investiguem a efetividade de sua aplicação com o público-alvo.

## Data Availability

Os dados de pesquisa estão disponíveis no corpo do artigo.
